# Subcutaneous Foslevodopa as Rescue Therapy for Abrupt Interruption of Oral Dopaminergic Treatment in Parkinson's Disease

**DOI:** 10.1002/mdc3.70253

**Published:** 2025-07-24

**Authors:** Fabienne Ory‐Magne, Jean Luc Houeto, Margherita Fabbri

**Affiliations:** ^1^ Department of Clinical Pharmacology and Neurosciences Toulouse Parkinson Expert Centre, Toulouse NeuroToul Center of Excellence in Neurodegeneration (COEN), French NS Park/F‐CRIN Network, University of Toulouse 3, CHU of Toulouse, INSERM Toulouse France; ^2^ Department of Neurology Limoges University Hospital, Inserm, U1094, EpiMaCT Epidemiology of Chronic Diseases in Tropical Zone Limoges France

**Keywords:** Parkinson's disease, foslevodopa infusion, rescue therapy, prevention of malignant syndrome

Abrupt withdrawal of antiparkinsonian medication in patients with Parkinson's disease (PD) can worsen motor symptoms and lead to serious complications, including dysphagia, aspiration, and malignant parkinsonian syndrome which can be at worse life‐threatening.^1^, ^2^ Sudden cessation of dopamine agonists may also trigger a withdrawal syndrome with psychiatric and autonomic symptoms.^3^ These interruptions often occur during periods of fasting, altered consciousness, gastrointestinal dysfunction, or perioperative care.^4^ Apomorphine, delivered via subcutaneous bolus or continuous infusion (CSAI), may serve as an effective alternative to maintain dopaminergic stimulation and prevent such adverse outcomes.^4^

Based on this rationale, we initiated emergency subcutaneous foslevodopa (FOS) infusions in four patients for whom oral administration was no longer feasible (Table 1). Infusions were delivered via a standard electric syringe pump, allowing us to circumvent the 48‐h procurement delay typically required for the Vyafuser® pump, the conventional delivery device for FOS. This approach also avoided pump wastage, as current traceability regulations prohibit the reuse of a Vyafuser® pump between patients. In the first two cases, the use of foslevodopa was anticipated to be temporary. In the third and fourth cases, treatment outcomes were uncertain, particularly given both patients’ history of severe visual hallucinations under CSAI. In the third case, prior pump interruptions (due to device malfunction, catheter dislodgement) or oral medication refusal had resulted in marked motor deterioration, including painful dystonia and loss of ambulation, placing the patient at high risk for rapid functional decline. In the fourth case, the patient was treated exclusively with apomorphine, raising concern for severe parkinsonian decompensation in the absence of an alternative. Following the discontinuation of both CSAI and oral levodopa, the initiation of FOS stabilized motor and behavioral symptoms in both patients, enabling transition to long‐term FOS therapy. The choice between daytime‐only and 24‐h FOS was tailored to each patient based on their clinical profile and symptom severity. Daytime infusion was used to avoid dopaminergic therapy interruption or with prior success using daytime‐only CSAI. In contrast, 24‐h infusion was employed for a patient with severe nocturnal akinesia and prior benefit CSAI. FOS dosing followed an individualized titration protocol, guided by levodopa equivalent dose (LED) calculations using Tomlinson's formula. Infusion rates were derived by converting daily LED to FOS volume (100 mg LED = 0.6 mL) and adjusting for infusion duration, with increments limited to 0.1 mL/h due to pump constraints. Nocturnal doses were capped at 0.2 mL/h in patient with hallucinations to mitigate neuropsychiatric risk. Dose escalation was gradual with adjustments every other day. After clinical improvement, pumps were customized for foslevodopa delivery.

**TABLE 1:** Patients characteristics

	Patient 1	Patient 2	Patient 3	Patient 4
Patients characteristics				
Age	48 years	78 years	67 years	76 years
Disease duration (years)	9	8	9	9
Hoehn and Yahr scale (On)	3	4	4	3
Hoehn and Yahr scale (Off)	5	5	5	5
MDS UPDRS 4.3 before FOS	0	0	4	4
MDS UPDRS 4.3 at the end of FOS test	1	0	2	2
Cognitive impairment with limitations of autonomy	No	Yes	Yes	No
Description of their treatment				
Antiparkinsonian drugs	Levodopa carbidopa Amantadine Ropinirole	Levodopa carbidopa	Apomorphine Levodopa carbidopa	Apomorphine
Number of doses of oral levodopa/day	5	5	7	0
Ongoing‐subcutaneous apomorphine infusion	No	No	Yes	Yes
Levodopa equivalent daily dose (mg) before FOS	1850	750	2040 (including 390 apomorphine LED)	700 mg (consisting in LED apomorphine only)
Clozapine before FOS	No	No	Yes	Yes
FOS treatment				
Causes of discontinuation of oral therapy	Vigilance disturbance after a fall with head trauma and severe rhabdomyolysis	Recurrent paralytic ileus in a patient with severe constipation and recurrent vomiting	Dopaminergic psychosis with refusal of oral treatment	Dopaminergic psychosis with refusal of oral treatment
Objectives of FOS administration	Optimize recovery and avoid nasogastric tube to administer parkinsonian treatment	Administer Parkinson's treatment as enteral administration was not possible.	Administer parkinsonian treatment as the patient has refused oral therapy and apo should be stopped.	Administer parkinsonian treatment as the patient has refused oral therapy and apo should be stopped.
FOS hourly dose in mL/H and number of hours of administration during the day	0.7 for 12 h (0)	0.4 for 12 h (0)	0.6 for 15 h	0.4 for 16 h (0)
(hourly dose of FOS in mL/H and number of hours of administration at night) at the end of the test			(0.2 for 9 h)	
LED with FOS (mg)	1400	666	1800	1066
Duration of treatment with FOS	9 days	7 days	Continued (Ongoing for 6 months)	Continued (Ongoing for 3 months)
Was Clozapine introduced with FOS	No	No	No but its dosage was increased	No but its dosage was increased
Outcome	Favorable	Favorable	Favorable	Favorable

*Note*: MDS UPDRS 4.3 time spent in the OFF time 0: Normal: No OFF time. (1) Slight: ≤ 25% of waking day. (2) Mild: 26–50% of waking day. (3) Moderate: 51–75% of waking day. (4) Severe: >75% of waking day.

Abbreviations: Apo, continuous subcutaneous apomorphine infusion; LED, levodopa equivalent dose.

Although clinical experience with FOS is currently more limited than with CSAI, this therapeutic option may be particularly valuable in acute care settings where oral dopaminergic therapy is interrupted.^5^ Delivery via a standard electric syringe pump may eliminate the need for nasogastric tube placement. While further studies are needed to validate these findings, our experience suggests that FOS infusion using an electric syringe pump merits consideration in surgical, emergency, or early post‐acute contexts. This strategy may prevent malignant‐like parkinsonian syndromes associated with abrupt withdrawal of chronic levodopa therapy and could also serve as a short‐term test phase prior to initiating long‐term infusion therapy in clinically fragile patients. Simple and standardized care protocols could be developed for non‐neurology teams to ensure continuity of dopaminergic treatment and avoid serious complications related to its sudden cessation.

## Author Roles

(1) Research project: A. Conception, B. Organization, C. Execution; (2) Statistical Analysis: A. Design, B. Execution, C. Review and Critique; (3) Manuscript Preparation: A. Writing of the first draft, B. Review and Critique.

F.O.M.: 1A, 1B, 1C, 3A, 3B.

M.F.: 3B.

J.L.H.: 3B.

## Disclosures

**Ethical Compliance Statement:** Patients included from our unit were informed about the objective of the study, and the collection of non‐opposition to the retrospective use of medical data was carried out according to French law, good clinical practice and General Data Protection Regulation (GDPR). Written informed patient consent was not necessary for this work. According to French law, the retrospective and observational nature of the study (data collection) did not require approval by an ethics committee. We confirm that we have read the Journal's position on issues involved in ethical publication and affirm that this work is consistent with those guidelines.

**Funding Sources and Conflicts of Interest:** No specific funding was received for this work. The authors declare that there are no conflicts of interest relevant to this work.

**Financial Disclosures for the Previous 12 Months:** FO‐M has served as an advisory board member or consultant or has received travel grants from AbbVie, Aguettant, Orphalan, Orkyn, Biogen, Adelia, NHC France, Convatec. She reports grants from Fondation Santé Service. JLH has served as an advisory board member or consultant or has received travel grants from AbbVie, Aguettant, Orkyn, Biogen, Medtronic France and NHC France. He reports grants from ANR. MF has served as an advisory board member or consultant from Bial and Orkyn. She received honorarium to speak from Bial, Orkyn, Abbvie and Everpharma.

## Data Availability

The data that support the findings of this study are available from the corresponding author upon reasonable request.
